# Environmental and Climatic Determinants of Molecular Diversity and Genetic Population Structure in a Coenagrionid Damselfly

**DOI:** 10.1371/journal.pone.0020440

**Published:** 2011-05-31

**Authors:** Maren Wellenreuther, Rosa A. Sánchez-Guillén, Adolfo Cordero-Rivera, Erik I. Svensson, Bengt Hansson

**Affiliations:** 1 Department of Biology, Lund University, Lund, Sweden; 2 Departamento de Ecoloxía e Bioloxía Animal, Universidade de Vigo, Pontevedra, Spain; The University of Queensland, St. Lucia, Australia

## Abstract

Identifying environmental factors that structure intraspecific genetic diversity is of interest for both habitat preservation and biodiversity conservation. Recent advances in statistical and geographical genetics make it possible to investigate how environmental factors affect geographic organisation and population structure of molecular genetic diversity within species. Here we present a study on a common and wide ranging insect, the blue tailed damselfly *Ischnuraelegans*, which has been the target of many ecological and evolutionary studies. We addressed the following questions: (i) Is the population structure affected by longitudinal or latitudinal gradients?; (ii) Do geographic boundaries limit gene flow?; (iii) Does geographic distance affect connectivity and is there a signature of past bottlenecks?; (iv) Is there evidence of a recent range expansion and (vi) what is the effect of geography and climatic factors on population structure? We found low to moderate genetic sub-structuring between populations (mean F_ST_ = 0.06, D_est_ = 0.12), and an effect of longitude, but not latitude, on genetic diversity. No significant effects of geographic boundaries (e.g. water bodies) were found. F_ST_-and D_est_-values increased with geographic distance; however, there was no evidence for recent bottlenecks. Finally, we did not detect any molecular signatures of range expansions or an effect of geographic suitability, although local precipitation had a strong effect on genetic differentiation. The population structure of this small insect has probably been shaped by ecological factors that are correlated with longitudinal gradients, geographic distances, and local precipitation. The relatively weak global population structure and high degree of genetic variation within populations suggest that *I. elegans* has high dispersal ability, which is consistent with this species being an effective and early coloniser of new habitats.

## Introduction

The spatial structuring of intraspecific neutral genetic diversity contains important information about both historical and current evolutionary processes. For example, extensive gene flow will constrain divergence by preventing local genetic differentiation, whereas reduced dispersal and philopatry are expected to cause genetic subdivision [1], [2]. Various factors can maintain neutral genetic diversity over large geographic areas, such as spatial distances between populations [3], physical barriers to gene flow [4], and habitat suitability and/or fragmentation [4], [5], [6]. Moreover, intrinsic life history traits of the species studied (e.g. dispersal and lifespan) affect population genetic structure and hence the geographic distribution of molecular diversity [7], [8]. The relative contribution of these different factors has been difficult to estimate in the past, but recent advances in statistical and geographic genetics now makes it possible to study these factors in more detail (e.g. [9], [10]).

Many species in Europe have wide-ranging geographic distributions and several studies have demonstrated geographic signatures of within species' genetic diversity (e.g. [11], [12], [13], [14]), often even over small geographic scales. Nevertheless, although a variety of factors have been put forward to explain the geographic structure of genetic diversity within species, only a few studies have explicitly tested the causal environmental factors behind these geographic patterns [15]. Evaluatingthe importance of different environmental factors is crucial since these factors often interact dynamically with each other, thereby confusing the spatial signatures of molecular differentiation. For example, a recent study by Kittlein and Gaggiotti[16] found that the interactions between various environmental factors can mask expected isolation-by-distance signatures that are often found in population genetic studies (e.g. [17], [18]). Thus, there is a clear need to more explicitly address the underlying environmental factors producing geographic patterns in the molecular structure of species.

**Figure 1:**
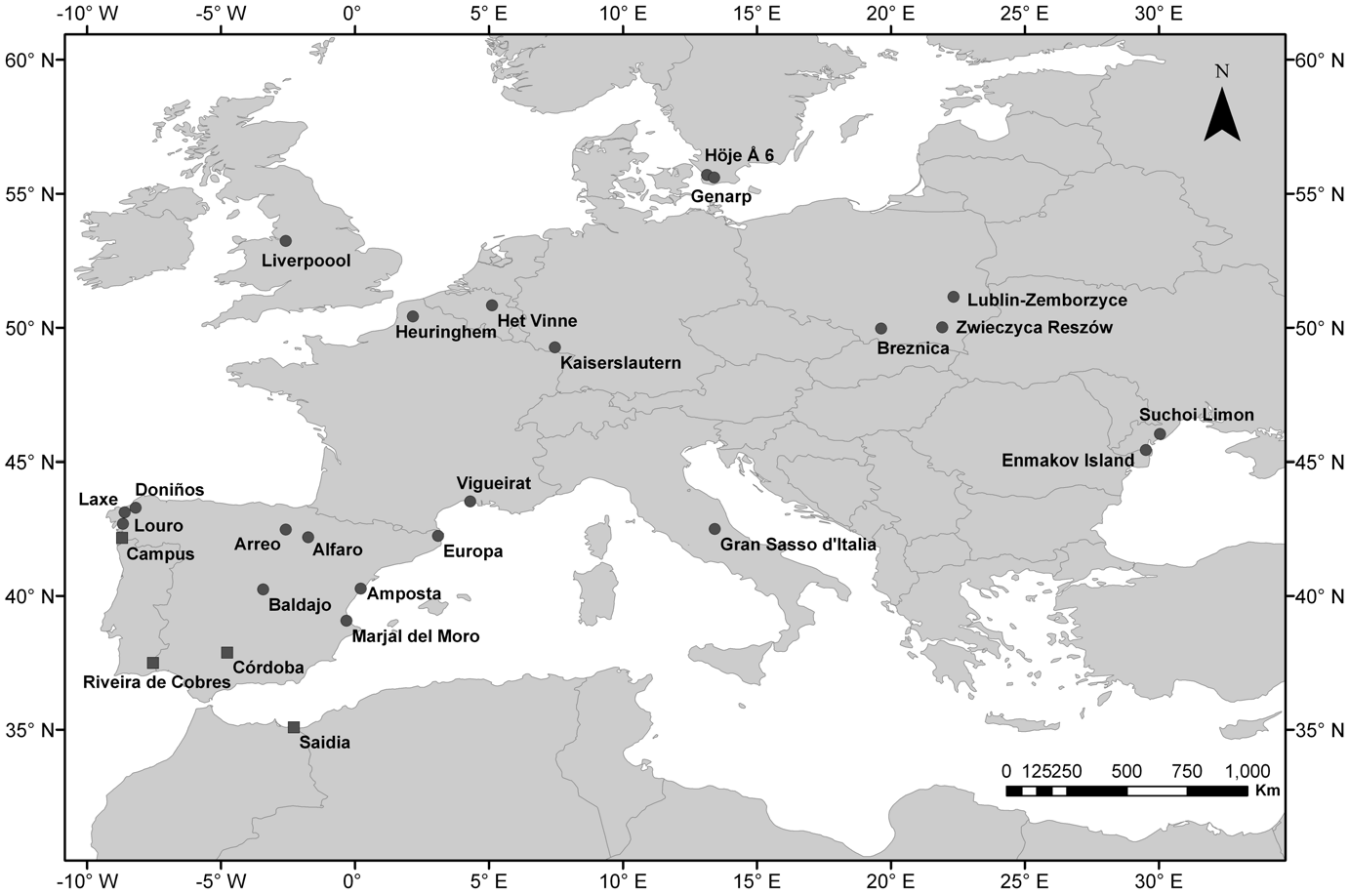
Map of *I. elegans* (n = 22) and *I. graellsii* (n = 4) study populations. The geographic range of *I. elegans* includes Europe with the exception of northern Scandinavia, Corsica, Sardinia, Sicily and Malta, and the western and southern parts of the Iberian Peninsula where it is replaced by its sister species *I. graellsii* [31]. The range of *I. elegans* further extends to the Middle East, and over much of Russia and China [31].

In this study, we investigated the genetic diversity and population structure of a common and wide-ranging insect, the blue tailed damselfly*Ischnuraelegans* (Odonata, Coenagrionidae). This small damselfly species is a well-investigated study system in evolutionary ecology, particularly in terms of mating interactions, sexual selection, female colour polymorphisms, frequency-dependent selection and sexual conflict [19], [20], [21], [22], [23]. Interest in this species has also arisen due to its enigmatic mating system and the presence of a heritable colour polymorphism in females [24], [25] and the rapid evolutionary dynamics that has been observed in natural populations [26], [27]. To investigate the geographic pattern of intraspecific genetic diversity of *I. elegans*, we investigated the molecular structure of 22 populations over most of the western part of this species' geographical range (spanning 12° in latitude and 38° in longitude; Figure 1), along with four populations of its congeneric sister species *I. graellsii*. These two sister species are similar in habitat choice and morphology [28], and hybridise in north-western Spain, where they produce fertile offspring [25], [28]. Analyses of DNA sequence variation of the mitochondrial *cytochrome b* and *coenzyme II* have shown that the genetic distance between *I. elegans*and *I. graellsii*is only 0.2%, suggesting that these two species are very closely related[25], or alternatively, that long-term on-going hybridization counteracts genetic divergence between *I. elegans* and *I. graellsii* [28], [29]. Molecular population diversity of both species was analysed with novel microsatellite markers that we specifically developed for *I. elegans.* Cross-amplification tests have revealed that these microsatellites are also polymorphic in *I. graellsii* [30]. The pattern of intraspecific genetic diversity in *I. elegans* was analysed with particular attention to longitudinal and latitudinal clines. We further investigated if geographic boundaries within the sampling area have led to discontinuities in molecular population structure, since both large water masses and mountains within the sampling area present potential barriers to dispersal. We also tested if geographic distance between populations exhibits an effect on population connectivity (i.e. dispersal) and investigated if we could find evidence for a signature of past historical bottlenecks. Finally, we evaluated several different ecological scenarios by relating environmental factors and their interactions to population specific F_ST_-values of *I. elegans*, namely the role of range expansion (latitude and longitude), geographic suitability (distance to coast and altitude) and climatic suitability (mean annual temperature and precipitation).

The results in this study suggest that this small insect has a weak genetic population structure across a major part of its geographic range, and that the genetic structure does not seem to be severely affected by large geographic barriers. Nevertheless, we found that high local precipitation rates (e.g. flooding events), which presumably reduce the local effective population size (N_e_:s), increased the degree of genetic differentiation of populations. Overall, these results confirm the emerging view that this species is a fast and efficient colonizer of disturbed habitats, and commonly undergoes population extinctions and re-colonisations [14].

## Materials and Methods

### Ethics Statement

All procedures were conducted according to the ethical guidelines of the relevant country to ensure ethical appropriateness, and catching permits were requested from the local authorities wherever necessary.

### Study populations and sample collection

Adults of the damselfly *I.elegans* were caught from 22 populations during the flying seasons 2005**–**2009 using hand nets. At each population, 11**–**20 (mean 17.4; median 18) damselflies were collected for subsequent genetic analysis (see Table 1 for details of each population). Sampling locations covered most of the western part of the distributional range of *I. elegans* [31] and spanned from 55° in the North, to 30° in the East, to 35° in the South, to -8 in the West (Figure 1). In addition, four populations of the sister species *I. graellsii* (total N = 66) were sampled in Spain (Campus Lagoas-Marcosende: 42°16′68N, 8°68′54W and Córdoba: 37°46′24N, 5°32′57W), Portugal (Riveira de Cobres: 37°29′45N, 7°31′12W) and Morocco (Saidïa: 37°49′60N, 7°52′00W) and kept for molecular analysis. Captured individuals were stored in 90% ethanol in small plastic tubes until DNA extraction. Additional sampling details are given in Table 1.

**Table 1:** Population details.

Species	Populations	Country	Region	Year	Latitude	Longitude	N	H_O_	H_E_	Alleles	Richness
*I. elegans*	Doniños	West Spain	South Europe	2007	43.29270	-8.18550	20	0.711	0.700	41	6.591
*I. elegans*	Laxe	West Spain	South Europe	2007	43.61930	-8.11126	14	0.715	0.805	32	6.146
*I. elegans*	Louro	West Spain	South Europe	2007	42.69088	-8.66035	15	0.712	0.729	32	5.792
*I. elegans*	Arreo	North Spain	South Europe	2008	42.47750	-2.57870	15	0.631	0.761	50	7.784
*I. elegans*	Baldajo	Central Spain	South Europe	2008	40.24260	-3.42060	17	0.603	0.795	48	7.673
*I. elegans*	Alfaro	North Spain	South Europe	2008	42.19080	-1.74230	20	0.663	0.758	50	7.046
*I. elegans*	Europa	East Spain	South Europe	2008	42.24380	3.10280	18	0.671	0.787	48	7.109
*I. elegans*	Amposta	East Spain	South Europe	2008	40.27320	0.21560	20	0.691	0.770	51	7.156
*I. elegans*	Marjal del Moro	East Spain	South Europe	2008	39.07270	-0.31350	20	0.671	0.751	44	5.776
*I. elegans*	Vigueirat	South France	South Europe	2008	43.53110	4.30120	16	0.733	0.804	42	6.252
*I. elegans*	Gran Sassod'Italia	Central Italy	South Europe	2008	42.50150	13.43280	19	0.777	0.813	51	7.461
*I. elegans*	Liverpool	Great Britain	North Europe	2008	53.24390	-2.58400	16	0.624	0.709	38	5.964
*I. elegans*	Heuringhem	North France	North Europe	2008	50.42090	2.16400	19	0.729	0.781	45	7.380
*I. elegans*	Kaiserslautern	South Germany	North Europe	2008	49.26410	7.46740	17	0.765	0.770	53	8.177
*I. elegans*	Het Vinne	Belgium	North Europe	2007	50.83300	5.11700	18	0.682	0.795	46	7.248
*I. elegans*	Höje Å 6	Sweden	North Europe	2005	55.70220	13.14370	20	0.653	0.717	43	7.010
*I. elegans*	Genarp	Sweden	North Europe	2005	55.60752	13.40420	20	0.680	0.753	44	7.203
*I. elegans*	Lublin-Zemborzyce	Poland	East Europe	2007	51.15000	22.34000	14	0.7505	0.797	60	8.081
*I. elegans*	ZwięczycaReszów	Poland	East Europe	2007	50.01670	21.91670	11	0.668	0.827	52	7.264
*I. elegans*	Breznica	Poland	East Europe	2007	49.96964	19.64290	18	0.712	0.796	47	6.678
*I. elegans*	Suchoi Limon	Ukraine	East Europe	2006	46.03000	30.04700	20	0.719	0.791	45	6.537
*I. elegans*	Enmakov Island	Ukraine	East Europe	2006	45.43500	29.52500	15	0.713	0.766	49	6.811
*I. graellsii*	Campus	West Spain	Outgroup	1999	42.166886	-8.68542	17	0.485	0.694	31	3.249
*I. graellsii*	Córdoba	South Central Spain	Outgroup	2005	37.883330	-4.76666	20	0.647	0.653	36	3.466
*I. graellsii*	Riveira de Cobres	Portugal	Outgroup	2009	37.49600	-7.52000	14	0.684	0.719	31	3.713
*I. graellsii*	Saidïa	North Morocco	Outgroup	2009	32.83000	-4.52000	13	0.490	0.677	25	3.118

Table shows the species, sampling localities, country, sampling year, latitude and longitude, the number of individuals sampled per population (N), observed (H_O_) and expected heterozygosity (H_E_), number of alleles and the allelic richness per population.

### DNA extraction and microsatellite genotyping

To extract DNA, the head of each damselfly was used, to avoid contamination with gut parasites and (or) sperm. Heads were subsequently dried and homogenized using a TissueLyser (Qiagen). DNA was extracted from the powder by proteinase K digestion followed by a standard phenol/chloroform-isoamylalcohol extraction [32]. The purified DNA was re-suspended in 100 µl of sterile water. The genotypes of all damselflies were assayed at six microsatellite loci previously isolated for this species [I-002, I-015, I-041, I-053, I-095, I-134, for details see 30]. These loci were previously described as being polymorphic with high heterozygosity (observed range: 0.46 to 0.88), and none of them was found to deviate from Hardy–Weinberg equilibrium or be in linkage disequilibrium with each other [30]. One primer of each pair was 5′-labelled with 6-FAM, HEX or NED florescent dyes. Polymerase chain reactions (PCRs) were carried out in 10 µL volumes on a GeneAmp PCR System 9700 (Applied Biosystems) and contained 4 pmol of each primer, 15 nmol MgCl_2_, 1.25 nmoldNTP, 0.5 U Ampli-taq polymerase and 10**–**20 ng template. The conditions were as follows: initial denaturation step of 94°C for 2 minutes, then 35 cycles at 94°C for 30 s, touch down from 62**–**58°C for 30 s, 72°C for 30 s followed by 72°C for 10 minutes. Multiplex primer reactions were performed for combinations of primers with matching annealing temperatures but differing size ranges and dye labels, then mixed with a labelled size standard and electrophoresis was conducted on an ABI PRISM 3730 Genetic Analyzer (Applied Biosystems). Resulting data were analyzed with GeneMapper 3.0 (Applied Biosystems) for internal standard and fragment size determination and for allelic designations. The same size standard was used on all samples analyzed for each marker.

### Population genetic analyses and geographic structure

Genetic diversity within populations was assessed in terms of allele frequencies, expected heterozygosity (H_E_), observed heterozygosity (H_O_), and allelic richness for each locus, using the program FSTAT version 2.9.3 [33]. Regression analyses of genetic diversity characteristics (allelic richness, number of alleles and heterozygosity estimates) and their associations with latitude and longitude were conducted to test for possible clinal relationships. In addition, the degree of genetic differentiation over all populations, as well as for each population pair, was estimated by calculating multilocus estimates of Weir and Cockerham's F_ST_ (θ). F_ST_ was used rather than R_ST_ [34], as it is considered a more reliable estimate of population differentiation when using relatively small data sets with fewer than 20 loci [35]. Significance of the global F_ST_-estimate was evaluated by permuting genotypes among samples and calculating 95% CIs by bootstrapping over loci (number of permutations was set at 1000). This method assumes Hardy-Weinberg equilibrium within populations. In the pairwise tests of population differentiation, the nominal statistical significance value of 5/1000 was adjusted for multiple comparisons using the Bonferroni correction when accounting for multiple testing to minimize type I errors.

In addition to F_ST_, Jost'sD_est_ was used as a measure of genetic differentiation between populations [36] and calculated for each population pair using the web based resource SMOGDv. 1.2.5[37]. D_est_ is a relative measure of differentiation, which ranges from zero (no differentiation) to one (complete differentiation), and simulations have shown that it is an unbiased estimator of differentiation, and outperforms F_ST_, over a range of sample sizes and for markers with different numbers of alleles (including highly variable microsatellite loci)[38]. We used 1000 bootstrap replicates and the harmonic mean of D_est_ across loci.

We used the Bayesian statistical framework provided by the program STRUCTURE version 2.2.3 [39] to analyse the geographic structure of the 22 *I. elegans* populations and the four *I. graellsii* populations, since a NJ tree (based on F_ST_-values between population pairs) did not result in a strongly supported tree (results not shown). STRUCTURE uses a Bayesian Markov chain Monte Carlo (MCMC) method to find the number of genetic clusters (each of which is characterized by a set of allele frequencies at each locus) that, based on the likelihood of the individuals' genotype to belong to these genetic clusters, minimizes deviations from Hardy–Weinberg equilibrium (HWE) and linkage disequilibrium (LD)[39]. Different admixture models are implemented in STRUCTURE [39], and because damselflies are known to be good dispersers, which would cause migration between populations, we used the ‘admixture model’ with ‘correlated allele frequencies’[40]. We did not use the sampling location of the individuals as a prior. For each model, a ‘burn-in’ period of 20,000 MCMC replicates and a sampling period of 100,000 replicates was used. We performed runs for a number of clusters (*K*), ranging from one to ten; and for each *K*, 20 iterations were run. In this way, multiple posterior probability values (log likelihood (lnL) values) for each *K* were generated, and the most likely *K* was evaluated by the *ΔK*-method following Evanno et al. [41]. This method compares the rate of change in lnL between successive *K*s and the corresponding variance of lnL of each *K* [41].

Clusters of **i**ndividuals were also inferred with the R-package [42] GENELAND 3.13 [43], which uses a Bayesian MCMC algorithm to cluster samples on the basis of both genetic and geographic information. Like STRUCTURE, GENELAND finds clusters by maximising HWE and minimising LD. However, spatial information of individuals is also accounted for at the Bayesian prior level in such a way that clusters corresponding to spatially organized groups are considered more likely than those corresponding to completely random spatial patterns. The benefit of using a spatial prior is to get more accurate inferences and to explicitly infer the spatial borders between inferred clusters. Due to substantial algorithm improvement in the recent versions of GENELAND software (from version 3.0.0 onwards), we used correlated gene frequency model that allowed us to detect subtle structures in the presence of low genetic differentiation [44]. Additionally, improvements in the post-processing scheme allowed estimation of the number of clusters (*K*), as well as the assignment of individuals to the inferred clusters in a single step, treating the number of clusters as unknown. The analysis was run to identify the geographic structures among populations and barriers to dispersal using (i) all 22 *I. elegans* populations and (ii) all 22 *I. elegans* populations and the four *I. graellsii* populations. To determine the number of genetic clusters, four independent runs were implemented for each analysis using 100,000 MCMC iterations with a burn-in period of 20,000 and a thinning value of 100 and then the model with the highest posterior probability was chosen. *K* was set to K_min_ = 1, K_initial_ = 4 and a K_max_ = 22 or 26 clusters, respectively, while filtering for null alleles during the run. It should be noted that the filtering was just a precautionary option, and that the model did not change when this option was not selected. However, this option allowed us to calculate the frequency of null alleles in our dataset. Consistent with previous results [30], the frequency of null alleles was very low for all loci (<0.002). The output map of the clusters from the analysis was then compared to geographic map to identify possible barrier to gene flow, which could, for example, be caused by mountain ranges or oceans.

To examine the distribution of the genetic variance among the clusters identified by GENELAND, an analysis of molecular variance (AMOVA) was conducted using ARLEQUIN version 3.11 [45]. Analyses of among-group variance were based on the five and six clusters identified by GENELAND, using the locus-by-locus settings for all analyses. The AMOVA program allows the hierarchical partitioning of the variance components into three components: among groups, among populations within groups, and among individuals within populations. Statistical significance was assessed by 10,000 permutations.

### Role of geographic isolation and bottlenecks

Isolation-by-distance, which is defined as a decrease in the genetic similarity among populations as the geographic distance between them increases [sensu 46], was investigated by correlating the pairwise differentiation (based on both F_ST_- and D_est_-values, but using F_ST_ /(1- F_ST_) and D_est_ /(1- D_est_), respectively [47]and geographical distances between *I. elegans* populations (i.e. logarithmic Euclidean distances between populations estimated using the GIS software ArcView 8.2, Environmental Systems Research Institute). To statistically determine if genetic and geographic distances between populations are significantly correlated, a Mantel test on the genetic and geographic matrix was performed (1,000 randomizations), using the program Isolation by Distance (Isolde) web service (http://ibdws.sdsu.edu/~ibdws/).

The program BOTTLENECK [48] was used to identify signals of recent bottlenecks. This program generates the expected heterozygosity under mutation-drift equilibrium (HetEQ) from the number of alleles at a locus and the sample size using the Stepwise Mutation Model (SMM), Two-Phase Model (TPM), and Infinite Allele Model (IAM), the HetEQ values are then averaged across loci and compared with the observed level of heterozygosity. The SMM and TPM are most appropriate for microsatellite data [49], with the TPM providing a more realistic picture of mutational events in microsatellite loci [48]. HetEQ was calculated using the SMM and the TPM, the latter allowing 95% single-step mutations and 5% multiple-step mutations (with a variance of the multiple steps of approximately 12%), following the recommendation of Piry et al. [47]. The program returns several nonparametric tests of whether heterozygosity deviates from that expected under HetEQ. The most powerful of these tests—and the one employed here—is the Wilcoxon test. This test is particularly appropriate when less than 20 loci are used [48].

### Range expansion, geographic suitability and climatic suitability

To identify the environmental factors that might determine the genetic population structure of *I. elegans*, we used the hierarchical Bayesian method of Foll and Gaggiotti[9] implemented in the programme GESTE. F_ST_-values are estimated for each local population (population specific F_ST_-values) and provide information on how genetically distinct a population is relative to other populations in the sample. For example, under a model of diffusive dispersal following a single colonization event, populations furthest from the origin would have the highest F_ST_-values due to the cumulative effects of drift from repeated founder events. Population-specific F_ST_-values were related to environmental factors using a generalized linear model. We chose this approach as our primary method because it tests multiple variables simultaneously. As suggested by the authors, we used the reversible jump MCMC method, and 10 pilot runs of a length of 5,000 as burn-in prior to drawing samples from a chain of 50,000 in length, separated by a thinning interval of 50. All combinations of variables were considered and models were evaluated using estimates of posterior probability, the 95% highest probability density interval (HPDI). The output also calculates the cumulative probability for each factor individually, so that the factor importance can be compared easily. GESTE can currently be run with two factors and their interaction at a time, and we run three different scenarios.

First, we investigated if there was any signature of gradual population expansion using the factors latitude and longitude in the analysis [9]. If a gradual population expansion has occurred, we can assume a fission model in which successive founder events would lead to a gradual increase in genetic differentiation between local and ancestral populations as the distance between them increases. Second, we investigated the role of geographical suitability by incorporating altitude and the distance to coast of each population as factors in the analysis. Finally, we investigated the role of local climatic factors by using the mean annual temperature and precipitation as factors in the analysis. These bioclimatic variables were extracted for each population in ARCGIS from the WorldClim climate data base (http://www.worldclim.org/bioclim).

## Results

### Population genetic analyses and geographic structure

Populations contained a substantial fraction of genetic variation, as shown by the pronounced genetic diversity at each locus (Table 1). Estimates of observed and expected heterozygosity were similar for the *I. elegans* populations and ranged from 0.60**–**0.77 and 0.70**–**0.83, respectively (Table 1). The total number of alleles over all loci ranged between 32 and 60 alleles among the populations studied. Estimates of allelic richness per locus were comparable between populations and ranged between 5.78**–**8.18 (Table 1).

The European populations of *I. elegans* were significantly differentiated from each other, although the differentiation was moderate to weak (global F_ST_ = 0.063, 95% CI 0.036**–**0.099, p<0.0001). All the investigated loci contributed to this population differentiation (each individual locus p<0.0001). The pairwise population differentiation ranged between F_ST_ = 0.00024 to F_ST_ = 0.14. Twenty-eight comparisons of these were statistically significant after applying a Bonferroni correction for multiple comparisons (p_Bonferroni_0.05_<0.00026; for details see Table 2). Some populations were genetically significantly distinct from a large number of populations. Specifically, the Spanish population Doniños, the two Polish populations (Lublin-Zemborzyce and ZwiêczycaReszów) and the two Swedish populations (Genarp and Höje Å 6) showed comparatively large and statistically significant genetic differences from several other populations (Table 2). F_ST_-values between *I. elegans* and *I. graellsii* populations ranged between 0.13 and 0.27 (Table 2).

**Table 2:** F_ST_-values (above diagonal) and statistical significance (below diagonal) between all study populations;the mean F_ST_value is 0.06.

Group	South Europe											North Europe						East Europe					Outgroup
Population	Doniños	Laxe	Louro	Arreo	Baldajo	Alfaro	Europa	Amposta	Marjal del Moro	Vigueirat	Gran Sassod'Italia	Liverpool	Heuringhem	Kaiserslautern	Het Vinne	Höje Å 6	Genarp	Lublin-Zemborzyce	ZwięczycaReszów	Breznica	Suchoi Limon	Enmakov Island	Igraellsii
Doniños		0.06	0.05	0.08	0.08	0.06	0.08	0.08	0.09	0.05	0.02	0.03	0.04	0.05	0.03	0.09	0.05	0.09	0.09	0.06	0.07	0.10	0.24
Laxe	X		0.08	0.08	0.06	0.07	0.07	0.06	0.08	0.06	0.04	0.07	0.06	0.07	0.04	0.09	0.08	0.08	0.06	0.06	0.06	0.09	0.13
Louro	X			0.14	0.09	0.12	0.06	0.10	0.12	0.09	0.05	0.08	0.05	0.07	0.06	0.10	0.09	0.10	0.12	0.08	0.11	0.10	0.16
Arreo	0.00024	X			0.01	0.01	0.03	0.00	0.00	0.01	0.07	0.05	0.05	0.04	0.04	0.06	0.04	0.09	0.06	0.06	0.04	0.10	0.27
Baldajo	0.00024	X	X	0.10048		0.00	0.02	0.00	-0.01	0.02	0.04	0.04	0.03	0.03	0.02	0.07	0.03	0.04	0.03	0.04	0.01	0.05	0.24
Alfaro	0.00024	X	X	0.28452	0.16071		0.04	0.01	0.00	0.03	0.05	0.05	0.05	0.05	0.02	0.08	0.05	0.06	0.05	0.05	0.01	0.07	0.24
Europa	0.00429	X	X	0.15976	0.05333	0.05619		0.00	0.02	0.03	0.04	0.04	0.01	0.02	0.01	0.02	0.03	0.04	0.04	0.02	0.02	0.06	0.23
Amposta	0.00024	X	X	0.49881	0.45190	0.10690	0.52071		-0.01	0.00	0.06	0.05	0.02	0.01	0.02	0.03	0.03	0.07	0.05	0.04	0.03	0.09	0.24
Marjal del Moro	0.00024	X	X	0.91810	0.89024	0.67833	0.37071	0.98810		0.03	0.06	0.06	0.04	0.04	0.03	0.07	0.05	0.06	0.05	0.05	0.03	0.08	0.25
Vigueirat	0.00167	X	X	0.23000	0.08690	0.00357	0.28238	0.18548	0.02714		0.04	0.04	0.02	0.01	0.02	0.03	0.02	0.09	0.06	0.06	0.04	0.10	0.24
Gran Sassod'Italia	0.01190	X	X	0.00048	0.00405	0.00119	0.01500	0.00167	0.00857	0.03262		0.02	0.02	0.03	0.00	0.07	0.04	0.03	0.04	0.02	0.03	0.04	0.20
Liverpool	0.00476	X	X	0.04500	0.03238	0.00048	0.01571	0.07667	0.00595	0.08333	0.06190		0.00	0.01	-0.01	0.04	0.00	0.06	0.06	0.03	0.05	0.06	0.27
Heuringhem	0.00024	X	X	0.06452	0.02690	0.00024	0.00905	0.28619	0.02476	0.03952	0.00357	0.26167		-0.01	-0.01	0.01	0.00	0.04	0.04	0.01	0.04	0.05	0.21
Kaiserslautern	0.00024	X	X	0.12190	0.03619	0.00024	0.01167	0.50857	0.00619	0.31286	0.00095	0.30857	0.42381		0.00	0.02	0.00	0.06	0.05	0.02	0.05	0.07	0.25
Het Vinne	0.00024	X	X	0.13500	0.10190	0.02452	0.16238	0.20643	0.14262	0.03571	0.03738	0.55738	0.84048	0.35905		0.02	0.00	0.02	0.02	0.00	0.00	0.03	0.21
Höje Å 6	0.00024	X	X	0.00238	0.00381	0.00024	0.00833	0.00810	0.00119	0.00786	0.00024	0.00571	0.18190	0.00143	0.03619		0.02	0.09	0.07	0.05	0.06	0.10	0.26
Genarp	0.00024	X	X	0.00857	0.01357	0.00024	0.00643	0.00095	0.00143	0.03071	0.00024	0.15500	0.18452	0.55405	0.18762	0.00524		0.05	0.05	0.02	0.03	0.07	0.25
Lublin-Zemborzyce	0.00024	X	X	0.00071	0.00452	0.00048	0.01262	0.00071	0.00786	0.00143	0.00024	0.00024	0.00024	0.00024	0.00429	0.00024	0.00024		0.00	-0.01	0.00	-0.01	0.24
ZwięczycaReszów	0.00024	X	X	0.00071	0.03333	0.00095	0.02429	0.00190	0.03881	0.00405	0.00024	0.00095	0.00214	0.00024	0.01119	0.00048	0.00024	0.74286		0.00	0.01	0.01	0.20
Breznica	0.00119	X	X	0.02690	0.05786	0.01024	0.04952	0.04690	0.14095	0.01595	0.00643	0.01500	0.01714	0.00595	0.01952	0.00238	0.00048	0.50286	0.55929		0.01	0.01	0.23
Suchoi Limon	0.00024	X	X	0.09857	0.31571	0.03214	0.01476	0.00048	0.24167	0.00786	0.00667	0.00381	0.00429	0.00119	0.11738	0.00024	0.00048	0.94571	0.69286	0.42333		0.01	0.23
Enmakov Island	0.00048	X	X	0.00214	0.02667	0.00310	0.03167	0.00214	0.03143	0.00643	0.00667	0.00310	0.00048	0.00190	0.00548	0.00024	0.00048	0.38667	0.22286	0.03262	0.12881		0.25
*I. graellsii*	X	X	X	X	X	X	X	X	X	X	X	X	X	X	X	X	X	X	X	X	X	X	

Adjusted nominal alpha level for multiple comparisons after Bonferroni correction was 0.00026 for table-wide significance (the populations Louro and Laxe were not included, as genotype data was missing at one locus). X denotes comparisons that were not carried out (due to missing data at one locus).

As mentioned above, we also calculated the D_est_-value for each population pair, since it represents an unbiased estimator of genetic differentiation [36]. The D_est_ measures of between population differentiation (Table 3) showed an overall similar pattern to the pairwise F_ST_-values (Table 2); however, the D_est_-values were on average slightly higher (mean D_est_across all population pairs was 0.12, while it was 0.06 for the F_ST_-values). The pairwise population differentiation ranged between D_est_ = −0.0085 to D_est_ = 0.5412 (Table 3). There was a high correlation between the pairwise D_est_- and F_ST_-values (Mantel test r^2^ = 0.80, p<0.001, 1000 randomisations). The main difference between the values was that overall differences increased, in particular the interspecific differences, when using the D_est_ formula (see Table 2 and 3). This suggests that the actual genetic differentiation (D_est_) between populations is actually higher than suggested using F_ST_-comparisons alone, and highlights the need to use the more unbiased estimation of D_est_ when evaluating the degree of differentiation between population pairs [38].

**Table 3:** D_est_ -values between all study populations;the meanD_est_value is 0.12.

Group	South Europe											North Europe						East Europe					Outgroup
Population	Doniños	Laxe	Louro	Arreo	Baldajo	Alfaro	Europa	Amposta	Marjal del Moro	Vigueirat	Gran Sassod'Italia	Liverpool	Heuringhem	Kaiserslautern	Het Vinne	Höje Å 6	Genarp	Lublin-Zemborzyce	ZwięczycaReszów	Breznica	Suchoi Limon	Enmakov Island	*I. graellsii*
Doniños		0.12	0.01	0.15	0.18	0.14	0.17	0.21	0.23	0.08	0.06	0.02	0.11	0.11	0.06	0.17	0.09	0.24	0.25	0.12	0.14	0.25	0.34
Laxe			0.14	0.21	0.20	0.18	0.23	0.20	0.30	0.24	0.13	0.23	0.23	0.22	0.14	0.27	0.24	0.28	0.19	0.22	0.17	0.31	0.12
Louro				0.24	0.24	0.26	0.19	0.30	0.27	0.25	0.07	0.15	0.12	0.21	0.14	0.20	0.25	0.27	0.42	0.21	0.25	0.28	0.31
Arreo					0.00	0.00	0.00	0.00	0.00	0.02	0.10	0.07	0.01	0.04	0.01	0.11	0.04	0.14	0.13	0.09	0.00	0.21	0.30
Baldajo						0.00	0.02	0.00	-0.01	0.01	0.11	0.10	0.03	0.07	0.03	0.18	0.08	0.07	0.11	0.11	0.00	0.12	0.42
Alfaro							0.06	0.01	0.00	0.05	0.10	0.13	0.08	0.09	0.04	0.13	0.08	0.14	0.17	0.12	0.01	0.19	0.28
Europa								0.00	0.00	0.04	0.07	0.10	0.02	0.06	0.01	0.06	0.08	0.05	0.13	0.06	0.02	0.13	0.39
Amposta									0.00	0.00	0.12	0.12	0.00	0.00	0.00	0.06	0.06	0.11	0.13	0.08	0.02	0.23	0.33
Marjal del Moro										0.01	0.06	0.12	0.03	0.03	0.03	0.12	0.10	0.09	0.11	0.10	0.01	0.13	0.37
Vigueirat											0.05	0.02	0.01	0.01	0.02	0.06	0.02	0.31	0.23	0.19	0.08	0.35	0.32
Gran Sassod'Italia												0.02	0.02	0.05	0.00	0.12	0.07	0.08	0.12	0.04	0.10	0.10	0.26
Liverpool													0.00	0.02	0.00	0.04	0.01	0.17	0.17	0.10	0.12	0.19	0.44
Heuringhem														0.00	-0.01	0.01	0.00	0.09	0.09	0.03	0.08	0.10	0.31
Kaiserslautern															0.00	0.02	0.00	0.14	0.14	0.04	0.13	0.17	0.42
Het Vinne																0.01	0.00	0.06	0.05	0.01	0.01	0.09	0.28
Höje Å 6																	0.02	0.15	0.13	0.08	0.10	0.17	0.48
Genarp																		0.12	0.11	0.04	0.10	0.15	0.43
Lublin-Zemborzyce																			0.00	0.00	0.00	0.00	0.54
ZwięczycaReszów																				0.00	0.02	0.01	0.41
Breznica																					0.01	0.01	0.46
Suchoi Limon																						0.03	0.35
Enmakov Island																							0.50

**Figure 2:**
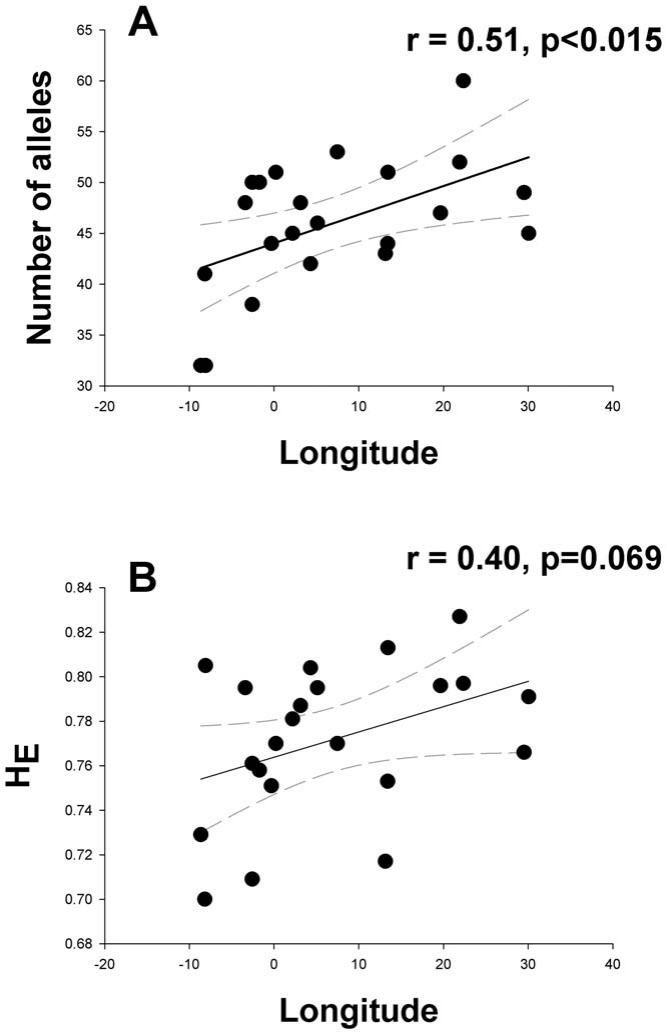
Linear regression between longitude and allelic richness of *I. elegans* populations (n = 22, r = 0.51, p<0.0015).

The geographic pattern of genetic variation measured as the number of alleles of *I. elegans* populations revealed a significant longitudinal cline (r = 0.51, r^2^ = 0.26, p<0.015; Figure 2A). There was a border-line significant relationship between longitude and expected heterozygosity (r = 0.40, r^2^ = 0.16, p = 0.069; Figure 2B). Regressions between longitude and observed heterozygosity and allelic richness were not significant, but both were positive in sign (r = 0.32 and r = 0.27, respectively). None of the regressions between genetic diversity and latitude were significant (p>0.05) and are therefore not shown.

**Figure 3:**
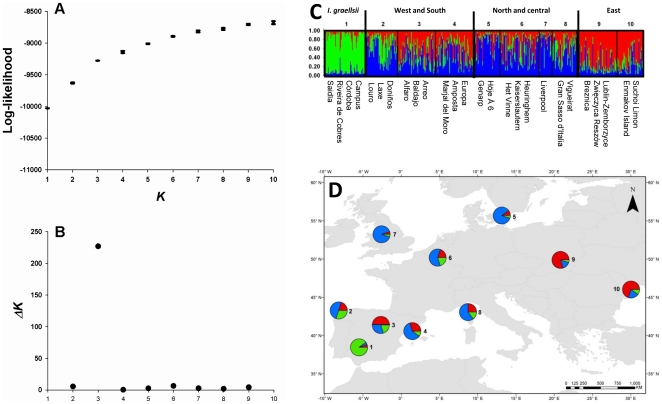
Estimated population structure of the 22 *I. elegans* populations and the four *I. graellsii* populations from Bayesian structure analysis using the program STRUCTURE 2.2.3. **A**). Mean likelihood (± SD) of *K* for different numbers of clusters **B**) *ΔK*-values for different *K*; suggesting *K* = 3 as the most likely structure according to Evanno et al. [41]. **C**) Individual Bayesian assignment probabilities for *K* = 3 for 22 populations of *I. elegans* and the outgroup*I. graellsii*(grouped for visualisation into ten geographically close groups). Individuals are represented by thin vertical lines, which are partitioned into *K* shaded segments representing each individual's estimated membership fraction. **D**) Pie charts show the mean membership fractions to each of the three genetic clusters in ten groups of populations.

To further evaluate intraspecific population differentiation between *I. elegans* populations, and their genetic similarity to *I. graellsii*, we used STRUCTURE to group populations into clusters. Structure supported the presence of differentiation among the populations, and the *ΔK*-method suggested three clusters as the most likely population structure (Figure 3A and B). The proportion of membership of each individual to each of the three genetic clusters (*K* = 3) is given in Figure 3C, and the average membership of individuals in closely located populations in 10 regions is given in Figure 3D. The proportion of membership of each individual to each of the 1**–**10 genetic clusters (*K* = 1**–**10) is shown in Figure S1. The results show a single very distinct *I. graellsii* group and three relatively diffuse genetic groupings of *I. elegans*that fall into a geographic pattern that consists of (i) northern and central (Sweden, Germany, Belgium, Great Britain, North France, South France and Italy), (ii) western and southern (Spain), and (iii) eastern populations (Ukraine and Poland; Figure 3C and D).

**Figure 4:**
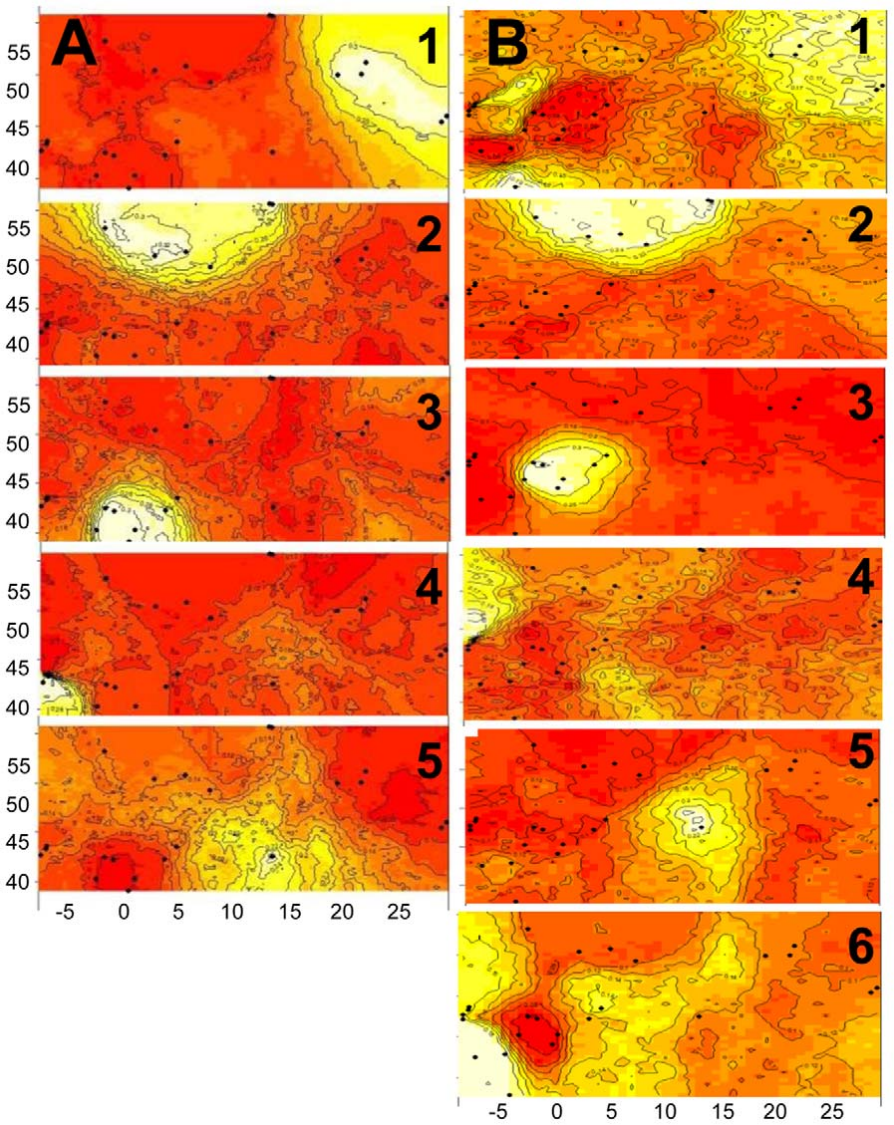
Spatial output from GENELAND using all 22 *I. elegans* populations (A) and (B) all 22 *I. elegans* populations and the four *I. graellsii* populations. Black circles indicate the relative positions of the sampled populations (see Figure 1). Darker and lighter shading are proportional to posterior probabilities of membership in clusters, with lighter (yellow) areas showing the highest posterior probabilities of clusters.

GENELAND was employed to complement the analyses run in STRUCTURE and to add a more explicit geographic component to the tests. Two analyses were run (22 *I. elegans* populations and 22 *I. elegans* populations and four *I. graellsii* populations)and these identified fiveand six clusters, respectively, of which the first five were identicalbetween analyses (Figure 4A and B). The first cluster contained all populations from Poland and the Ukraine (five populations), the second cluster consisted of populations from Germany, the UK, Sweden, northern France and Belgium (six populations), the third cluster contained populations from eastern Spain and southern France (seven populations), the forth cluster was made-up of populations from western Spain (three populations), and the fifth cluster consisted of the single Italian population(Figure 4A and B). The sixth cluster of the second analysis (22 *I. elegans* populations plus four *I. graellsii* populations) contained the four *I. graellsii* populationsin western andsouthern Spain and Morocco (Figure 4A and B). Finally, the finding that GENELAND identified a greater number of clusters than STRUCTURE (five/six versus three), and that the same clusters were identified by independent GENELAND runs and produced similar values of posterior probabilities, could indicate that the algorithm employed in GENELAND may be more sensitive to find weak clusters in space.

The genetic variance between thefive *I. elegans*clusters was quantified using an AMOVA. The major part of molecular genetic variation was found within populations (92.60%) with 4.30% among the five groups and 2.74% among the populations within groups (Table 4). Exact tests showed significant genetic variance on all these three levels (all three comparisons p<0.0001). We also quantified molecular variance between the six *I. elegans*clusters and the one*I. graellsii*cluster. The molecular variance within populations then decreased to 91.20%, and was still highly significant (Table 4). Genetic variance among groups increased to 6.17%, and the variance among populations within groups decreased slightly to 2.63% (Table 4).

**Table 4:** Analysis of molecular variance (AMOVA) based on six microsatellite loci.

(A)
Genetic variance	Among groups	Among populations within groups	Within populations
Five *I. elegans*clusters (22 populations), as identified by GENELAND	4.30***	2.74***	92.06***

(B)
Genetic variance	Among groups	Among populations within groups	Within populations
Five *I. elegans*groups and the one *I. graellsii*cluster (26 populations), as identified by GENELAND	6.17***	2.63***	91.20***

Significance levels are indicated (***: p<0.0001).

### Role of geographic isolation and bottlenecks

**Figure 5:**
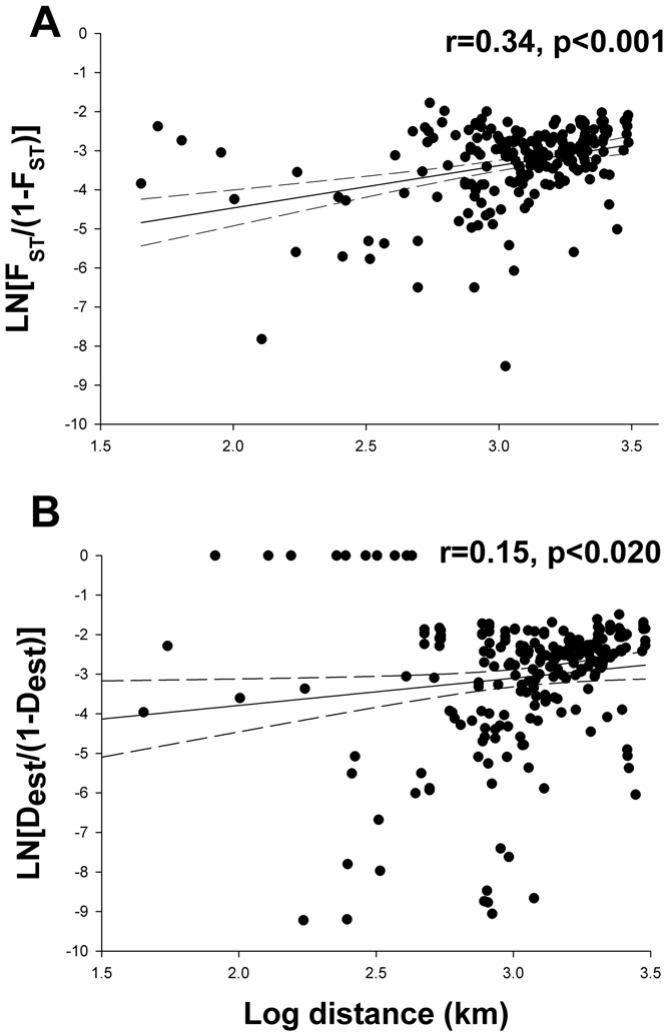
Relationship between pairwise F_ST_-values and the geographical distances for the 22 *I. elegans* populations. Test of isolation-by-distance: r = 0.34 and p<0.001. B) Relationship between pairwise D_est_-values and the geographical distances for the 22 *I. elegans* populations. Test of isolation-by-distance: r = 0.15 and p<0.020.

We tested for a possible pattern of isolation-by-distance between all population pairs (n = 22) of *I. elegans*. Applying a Mantel test to statistically investigate if the pair-wise matrix of genetic differentiation (F_ST_/(1-F_ST_) and D_est_/(1-D_est_), respectively) is correlated with the matrix of geographic distances, we did indeed find that the genetic population differentiation followed an isolation-by-distance pattern (F_st_: r = 0.34, one sided Mantel test p<0.001; D_est_: r = 0.15, p<0.02; Figure 5).

The program BOTTLENECK showed that only one of the populations examined (Laxe, p<0.047) showed a heterozygosityexcess, while four of the populations (Europa, Höje Å 6, Kaiserslautern and Marjal del Moro) showed a heterozygositydeficiency (Table 5). This suggests that some of the *I. elegans* populations show a weak signal of a heterozygositydeficiency, suggesting that they are not at a mutation–drift equilibrium, but that there has been a recent expansion in population size or a recent influx of rare alleles from genetically distinct immigrants. This trend is also supported by the overall lower mean of the heterozygositydeficiency compared to the heterozygosityexcess for all populations, which was 0.3 and 0.8, respectively.

**Table 5:** Test results from the program BOTTLENECK.

Populations	1-tail, heterozygosity-deficiency	1-tail, heterozygosity-excess	2-tail, both outcomes
Doniños	0.281	0.781	0.563
Laxe	0.969	**0.047**	0.094
Louro	0.078	0.953	0.156
Arreo	0.219	0.922	0.438
Baldajo	0.281	0.781	0.563
Alfaro	0.219	0.922	0.438
Europa	**0.040** *	0.977	0.078
Amposta	0.344	0.719	0.688
Marjal del Moro	**0.008** *	1.000	**0.016**
Vigueirat	0.500	0.578	1.000
Gran Sassod'Italia	0.422	0.656	0.844
Liverpool	0.055	0.961	0.109
Heuringhem	0.055	0.961	0.109
Kaiserslautern	**0.008** *	1.000	**0.016**
Het Vinne	0.922	0.219	0.438
Höje Å 6	**0.016** *	0.992	**0.031**
Genarp	0.078	0.945	0.156
Lublin-Zemborzyce	0.055	0.961	0.109
ZwięczycaReszów	0.500	0.578	1.000
Breznica	0.078	0.945	0.156
Suchoi Limon	0.344	0.719	0.688
Enmakov Island	0.055	0.961	0.10938

*bold *P*<0.05 (rejection of null hypothesis of mutation drift equilibrium).

Table shows the results for testing the null hypothesis for mutation drift equilibrium under the two phase model (TPM, 95% single-step mutations and 5% multiple-step mutations) using the Wilcoxon test.

### Range expansion, geographic suitability and climatic suitability

**Figure 6:**
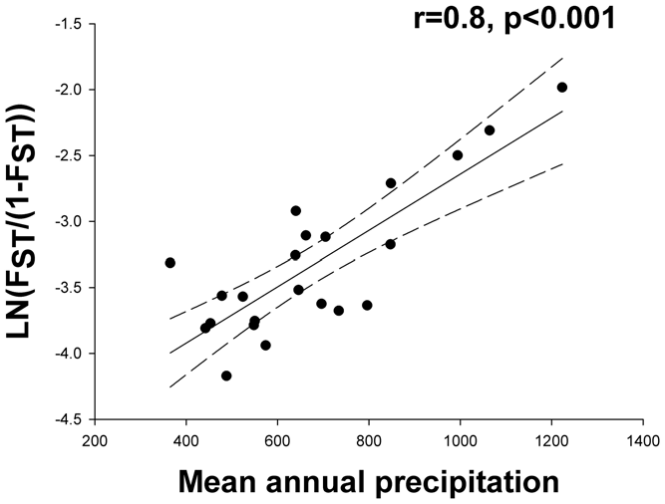
Relationship between the population specific F_ST_-values and mean annual precipitation at each population (see **Table 5** and Results for additional statistics).

When testing for the possible signature of a recent range expansion in GESTE, no effect of latitude or longitude on the population-specific genetic differentiation could be detected, thus rejecting a model of gradual range expansion in this species. The model including longitude and the constant had the second highest posterior probability (0.108), while the model containing latitude and the constant achieved a much lower posterior probability (0.047). The finding that longitude (east**–**west) was also more important than latitude (south**–**north) was further supported when looking at the data fit with just the factors alone, which resulted in a posterior probability of 0.117 and 0.056, respectively. Similarly, neither the distance to coast or altitude (geographic suitability) was strongly correlated to the population-specific F_ST_-values. Out of the two variables, the model including the constant term and distance performed better than the model containing the constant and altitude (0.133 and 0.058, respectively). In both of these aforementioned tests (range expansion and geographic suitability), the model that only included the constant term had the highest posterior probability (0.835 and 0.801, respectively, see Table 6). This means that in each of the two analyses, the model excluding all variables had at least an 80% probability of being the one that best fits the genetic structure observed. When testing for the climatic suitability, however, the model including the constant term and mean annual precipitation had the highest posterior probability and lowest variance and was thus deemed the best model (0.824, modal value 0.448, 95% HPDI 0.184 and 0.769, Table 6). The inclusion of the mean annual temperature did not improve the model fit (all models including this term had a posterior probability of <0.05). Adding temperature to the model with the constant term only reduced the posterior probability, again suggesting that this term has much weaker influence on the local genetic differentiation than precipitation (Table 6). All models that did not include the precipitation factors had a much lower posterior probability than models including precipitation. The regression coefficient for precipitation was positive, revealing that the population-specific F_ST_-values will be higher in areas where precipitation is high (see Figure 6).

**Table 6:** Posterior probabilities for different models (2 factors with their interaction) under the three environmental scenarios from the GESTE analysis.

Environmental Scenario	Factors	Posterior probability
Spatial range expansion	**Constant**	**0.835**
	Latitude	0.0563
	Constant, Latitude	0.0469
	Longitude	0.117
	Constant, Longitude	0.108
	Constant, Latitude, Longitude	0.00940
	Constant, Latitude, Longitude, Latitude*Longitude	0.00120
Geographic Suitability		
	**Constant**	**0.801**
	Altitude	0.0644
	Constant, Altitude	0.0579
	Distance to Coast	0.140
	Constant, Distance to Coast	0.133
	Constant, Altitude, Distance to Coast	0.00650
	Constant, Altitude, Distance to Coast, Altitude * Distance to Coast	0.00100
Climatic Suitability		
	Constant	0.116
	Temperature	0.0496
	Constant, Temperature	0.00570
	Precipitation	0.867
	**Constant, Precipitation**	**0.824**
	Constant, Temperature, Precipitation	0.0434
	Constant, Temperature, Precipitation, Temperature * Precipitation	0.0114

## Discussion

### Population genetic analyses and geographic structure

F_ST_-values between *I. elegans* populations were generally quite low (mean F_ST_ = 0.06), and the D_est_-values (meanD_est_ = 0.12) of the pairwise genetic population differentiation, albeit higher, were also low to moderate. Together these results suggest a relatively high degree of genetic connectivity across the species' geographic range in Europe, or alternatively, a recent population expansion. Odonates (dragonflies and damselflies) are thought to be relatively good dispersers, and often leave their natal habitat after emergence in the search for new ponds and/or rivers [50], [51]. Small-scale dispersal also occurs during the aquatic life-stage of odonates[52], but the realized amount of dispersal during this stage is challenging to reliably quantify. Several species in the genus *Ischnura* are known to be good dispersers, as their presence in remote archipelagos demonstrates [53]. *Ischnuraelegans*has been described as an opportunistic damselfly species that is typically found in quite disturbed environments, such as human-made artificial ponds [27] and can, unlike many other odonates, tolerate most plants as perching substrate [54]. Given that *I. elegans* exists in environments that experience strong temporal and spatial heterogeneity, leading to strong fluctuations in local population densities, the species experiences large fluctuations in both the strength and direction of selection. This is probably partly the reason for why local populations go extinct at a high rate, i.e. there is high population turnover in this species. Some of the data in this study (e.g. the relatively low D_est_-values and the diffuse population structure across large ranges) also support the general picture that *I. elegans* is an opportunistic insect species that rapidly colonises newly created habitats [55], but which has low local population persistence and is a weak competitor against other odonates. Presumably, other small coenagrionid damselflies have similar high dispersal potentials as *I. elegans*. *Ischnurahastata*, for example, is one interesting species in this respect, as it has been captured on nets mounted on airplanes and has also colonised the Galapagos islands [56]. It should be mentioned that the individual sample size per population in our study ranged between 11**–**20 individuals(mean 17.3, median 17.5, including the four *I. graellsii* populations), which is lower than the recommended sample size for stable F_ST_- and D_est_-estimates. Despite this shortcoming, we would like to highlight that the strength of our study laid in the high number of populations analysed and the large geographic area covered, which allowed us to investigate large scale environmental patterns and clines.

Molecular studies on other odonate species show a higher degree of genetic differentiation.For example, a study by Keller et al. [12] on the lilypad whiteface dragonfly *Leucorrhiniacaudalis* shows a slightly higher degree of microsatellite differentiation (F_ST_ = 0.130) between populations in Switzerland, and a study on the southern damselfly *Coenagrionmercuriale* by Watts et al. [57] in the UK found also a higher F_ST_-value of 0.17. The two aforementioned studies covered a much smaller geographic area than the present study and are both relatively rare and threatened species, unlike *I. elegans*.The F_ST_-values for these two rarer species strengthens our conclusionsthat the more abundant and dispersive species*I. elegans*consists ofpopulations that are connected by a high degree of gene flow, even over large geographic areas, or has been recently expanding in the area. A third study by Watts et al. [58]on the small red-eyed damselfly *Erythrommaviridulum*reports similarly low F_ST_-values as in our study, and this study was carried out on a large geographic scale, including samples from the UK, Germany, Netherlands, Belgium and France. Watts et al [58], [59]came to a similar conclusion to our study, namely that *E. viridulumappears* to be capable of relatively long distance dispersal, even over inhospitable habitat. *Erythrommaviridulum*is also a species that is common and expanding northwards, including recent establishment in southern Sweden, and has thus a similar ecology as *I. elegans*, compared to the aforementioned rarer species with more fragmented and localized populations.

Populations that contributed most to significant between-population differences were found at the edge of the sampling range (Table 2). These included populations in south-western Europe (Spain: Doniños), eastern Europe (Poland: Lublin-Zemborzyce, and ZwięczycaReszów) and northern Europe (Sweden: Genarp and Höje Å 6, Table 1). Of these, the south-western and northern populations can be defined as peripheral populations while the eastern range extends all the way to China [31]. Thus, the Polish populations should not be considered as peripheral, but are rather central populations. Peripheral populations are expected to show increased inter-population differentiation due to lower effective population sizes (N_e_) and concomitant increased potential for genetic drift [60], [61]. Such isolated populations also suffer restricted gene flow with other isolated marginal populations [62], [63]. If populations at the edge become more or less isolated from gene flow with the central area, then genetic drift and the associated loss of genetic information is expected to play an even stronger role [64]. A major goal in future research would be to understand how local population dynamics in *I. elegans* affect gene flow and how this interacts with the selection regimes experienced at the edge of their range. Although microsatellite loci are not directly under selection, due to the fact that they are non-coding genes, strong local selection at range limits [c.f. 65] would be expected to lower the effective population sizes and hence increase the potential for genetic drift [66]. In addition, asymmetrical gene flow from the centre of the range can limit or prevent adaptation of populations at the periphery, even if the latter experience intense directional selection [64], [65]. However, we would like to underscore that this hypothesis needs to be investigated using quantitative genetic data from adaptive traits and experiments (e.g. reciprocal transplants), and it cannot be addressed using only neutral markers [62], [63].

Genetic differentiation is thought to reflect the interplay between stochastic and selective factors that jointly influence the realised amount of population differentiation. In the case of *I. elegans*, it is likely that environmental gradients (e.g. in temperature and precipitation) together with fluctuations in population size (due to stochastic events and habitat fragmentation) are responsible for the heightened genetic differentiation of peripheral populations relative to the rest of the populations (Table 2). Moreover, the previously documented on-going hybridization between *I. elegans* and *I. graellsii* in Spain [25], [28] could potentially affect the degree of genetic differentiation of the Spanish *I. elegans* populations versus the other *I. elegans* populations in Europe [67]. Our statistical analyses provided evidence for a significant longitudinal cline of genetic diversity between *I. elegans* populations (Figure 2), while we found no evidence for latitudinal clines. It should be noted, however, that the latitudinal range that was covered in the present study (central Spain to southern Sweden) spans a much smaller geographic area than the covered longitudinal range (western Spain to eastern Europe), thereby making it less likely for latitudinal clines to occur in our material. Nevertheless, we conclude that the evidence in our study for a longitudinal cline is a robust result that deserves attention in future studies investigating *I. elegans*. Longitudinal gradients in genetic diversity in Europe have been less frequently reported than latitudinal gradients, and have typically been associated with postglacial colonization processes [68], [69], [70]. In our study, the longitudinal pattern of genetic diversity might indicate a post-glacial westward expansion from eastern refugia, but more data need to be collected to explicitly test this hypothesis. A postglacial westward range expansion was recently suggested for the Italian agile frog *Ranalatastei* [71], whereas an eastward range expansion was suggested for the great read warbler *Acrocephalusarundinaceus* [72].

The STRUCTURE results indicated weak divisions between southern and central, northern, and eastern population clusters of *I. elegans* (Figure 3), and the results from the spatial clustering analyses conducted in GENELAND suggested that the GENELAND algorithm was more powerful to detect genetic clusters than STRUCTURE (Figure 4). This could be due to the fact that STRUCTUREonly uses individual multilocus genotype data to infer population structure, while GENELANDalso exploits the spatial positions of the individual samples as a supplemental parameter in the analyses. Using the same dataset as in STRUCTURE (22 *I. elegans* and four*I. graellsii* populations), we were able to detect six clusters (Figure 4) (instead of three in STRUCTURE; Figure 3). Comparing these geographic clusters to geographic features (such as water bodies and mountains, which would clearly constitute significant barriers to dispersal for damselflies) did not highlight any clear geographic boundaries to gene flow. Instead, the geographic location of clusters appeared to be largely independent of potential barriers to dispersal. This suggests that both large water bodies (the North and Baltic seas for instance) or mountains (such as the Carpathian mountain range in the Ukraine and Poland) are unlikely to constitute major barriers to dispersal for *I. elegans* or, alternatively, that *I. elegans* can easily use other corridors to colonise habitats that are surrounded or close to such geographic structures.

Based on the clusters identified by GENELAND, we partitioned the molecular variance within and between all*I. elegans*populations and also within and between all *I. elegans*and the *I. graellsii*populations (Table 4). The analyses suggest a general high level of intrapopulation variation in *I. elegans*, indicating that this species is associated with large population sizes and/or frequent exchange of individuals between populations, which contrasts the pattern of reduced levels of intrapopulation genetic variation that has been found in other species that have expanded their range after the last Pleistoceneglacialmaxima (e.g. [68], [69], [70], [73]).

### Role of geographic isolation and bottlenecks

The genetic differentiation between *I. elegans* populations in Europe showed a clear geographic signature of isolation-by-distance (Figure 5).Abbott et al. (2008) did not find any significant isolation-by-distance in their study of a geographically much more restricted set of *I. elegans*populations in southern Sweden (maximum distance between populations  = 20 km). The absence of any significant pattern of isolation-by-distance in their study might indicate a relatively low degree of statistic power to detect a geographic signature in their case due to the small-scaled nature of their study, possibly in combination with the fact that these northern marginal populations might not be in equilibrium [26]. The pattern of isolation-by-distance in our larger geographic study area, in combination with relatively few loci genotyped, may further explain why the Bayesian clustering approach implemented in STRUCTURE found support for few distinct clusters and a rather diffuse population structure [39]. This problem was reduced in GENELAND (Figure 4), presumably because spatial geographic information was also utilised.

Analyses using BOTTLENECK did not provide strong support that any of the populations suffer from an excess or deficiency of heterozygosity. The only population to show a heterozygosity excess was the Spanish population Laxe. In another study (R. Sanchez-Guillen et al., unpublished), we have found that out of all populations examined for Spain, Laxe showed the highest degree of hybridization between *I. elegans* and *I. graellsii*, which could explain the excess of heterozygosity detected for this population. Apart from this population, there was a slight trend indicating that four populations showed a heterozygositydeficiency. Nevertheless, although the low power of this result prevents to make any strong statements, the result could point towards a situation where these populations have recently expanded in size.

The emergence of population bottlenecks is probably counteracted by the high dispersal potential in *I. elegans*, as it enables the rapid colonisation of new areas and also maintains gene flow between populations. The ability to disperse and colonise novel habitats is particularly important when the natal habitat becomes unsuitable, for instance, as a result of habitat deterioration or due to climate change [74]. Increasing temperatures have indeed been suggested to facilitate range expansion northwards in several ectotherms and insect species (e.g. [74], [75], [76]). For example, out of 35 butterfly species in Europe, 22 have shifted their ranges northwards by 35–240 km over the last century, whereas only two have shifted south [77]. A recent study on odonate range expansions in the UK showed that *I. elegans* has expanded its range 168 km northwards in the last few decades, which is more than double the average distance found for other odonate species in the same study [78]. This recent range expansion of *I. elegans* in the UK further demonstrates that *I. elegans* has the ability to quickly respond to environmental changes by dispersing to new areas. This suggests that the terrestrial adult phase in odonates plays a crucial role in genetically homogenizing closely as well as quite distantly located populations.

### Range expansion, geography and climatic suitability

We evaluated three different scenarios to identify environmental factors that potentially affect the genetic population structure of *I. elegans*, each of which included two factors (Table 6). The program GESTE calculates population-specific F_ST_-values (i.e. differences between one population versus the pool containing all other populations) and correlates these differentiation values to the environmental factors. The first scenario was to test if the inclusion of latitude and/or longitude in the model would result in a higher posterior probability than when the model was run without these factors, thereby identifying any signatures of spatial population processes, such as range expansions. A recent range expansion would partly account for the relatively low levels of population differentiation that we detected in *I. elegans*, since a recent expansion from a large ancestral population andthe retention of ancestral polymorphisms would be expected to lower the overall population differentiation [69], [70]. However, despite the plausibility of this scenario, the model statistically rejected the possibility of a gradual range expansion (from east to west, or south to north). We were also able reject the geographic suitability model, which included altitude and distance to coast as the explanatory factors. Finally, by including two measures of climatic suitability (mean annual temperature and precipitation) we found that, although temperature did not improve the model fit, precipitation had a large and significant effect on the genetic population differentiation in *I. elegans* (Figure 6). The positive regression coefficient for precipitation is consistent with the expectation that F_ST_-values will be higher in areas of higher precipitation because water bodies in such areas exhibit a greater magnitude and frequency of flooding. Higher frequencies of intense flooding are likely to degrade suitable habitat for both larvae and adults, thereby causing a decreases in the effective population sizes. The finding that precipitation can have a large and negative effect on the survival of odonates is supported by a study on the damselfly *Pyrrhosomanymphula*byGribbin and Thompson [59], which shows that the percentage mortality of this species was significantly and positively correlated with precipitation. Moreover, high rain fall during prolonged periods reduces the available time during summer to forage, mate and reproduce and could potentially contribute to local population extinctions in some years and areas (E. I. Svensson, personal observations). A negative effect on population persistence is likely to be particularly strong for a small species like *I. elegans*, which should make it particularly vulnerable to starvation. Thus, local extinctions, or a reduction in population sizes, are likely to be more frequent in areas that experience a significantly higher rate of precipitation. The influence of climate-related factors, such as precipitation, on the population structure and species diversity is of growing interest in conservation due to the possible impacts of climate change [74], [79], [80]. It should be noted, however, that climatic factors, such as precipitation, are likely also correlated with other environmental variables, which could have caused the positive relationship.

In conclusion, the present-day structure of *I. elegans* is likely to have been shaped by several ecological factors, including good dispersal ability and high temporal and spatial turnover of peripheral populations, making this species a good coloniser of newly established and disturbed habitats. We found that although the geographic distance affects the connectivity between populations, gene flow does not seem to be strongly affected by major geographical barriers to dispersal, such as seas and mountains. These factors are probably the main explanation for an overall weak global population structure and high degree of genetic variation within local populations. We also found a longitudinal population genetic signature, and that precipitation had a significant effect on the genetic differentiation of populations, in this species. These results suggest that longitudinal environmental gradients have resulted in genetic clines, and that the local flooding and drying sequence affects overall genetic differentiation. In recent years, *I. elegans* has significantly extended its range [78], which is consistent with a response to increasing regional temperatures in Europe [80]. Given that many aspects of *I. elegans*' ecology have been thoroughly investigated in recent past, this species can become an interesting model organism to understand how insects can cope with on-going climate and environmental change.

## Supporting Information

Figure S1 **Individual Bayesian assignment probabilities for** ***K*** **1–10 using the program STRUCTURE 2.2.3 for populations of** ***I. elegans*** **and** ***I. graellsii*** **.** Individuals are represented by thin vertical lines, which are partitioned into *K* coloured segments representing each individual's estimated membership fraction.
(TIF)
