# Supplementary figures and images for: Environmental and Climatic Determinants of Molecular Diversity and Genetic Population Structure in a Coenagrionid Damselfly
Source: PLoS One. 2011 May 31;6(5):e20440. doi: 10.1371/journal.pone.0020440 (PMC3105071; doi:10.1371/journal.pone.0020440)

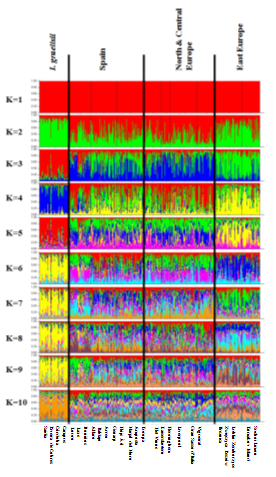

Supplement: Figure S1 — Individual Bayesian assignment probabilities for K 1–10 using the program STRUCTURE 2.2.3 for populations of I. elegans and I. graellsii . Individuals are represented by thin vertical lines, which are partitioned into K coloured segments representing each individual's estimated membership fraction. (TIF) [file pone.0020440.s001.tif]
